# Daily or Nondaily Vaping and Smoking Cessation Among Smokers

**DOI:** 10.1001/jamanetworkopen.2025.0089

**Published:** 2025-03-05

**Authors:** Natalie E. Quach, John P. Pierce, Jiayu Chen, Brian Dang, Matthew D. Stone, David R. Strong, Dennis R. Trinidad, Sara B. McMenamin, Karen Messer

**Affiliations:** 1 Herbert Wertheim School of Public Health and Human Longevity Science, University of California, San Diego, La Jolla; 2 Moores Cancer Center, University of California, San Diego, La Jolla

## Abstract

**Importance:**

An important public health goal is to increase tobacco cessation, but there is limited research on associations of vaping with tobacco cessation.

**Objective:**

To estimate the association of vaping with long-term tobacco cessation among US cigarette smokers who used electronic nicotine delivery systems (ENDS; ie, e-cigarettes) in 2017.

**Design, Setting, and Participants:**

This cohort study used a nationally representative sample of US cigarette smokers from the Population Assessment of Tobacco and Health cohort at wave 4 (2017) with follow-up at wave 6 (2021). Data were analyzed from June 2023 to June 2024.

**Exposure:**

Wave 4 ENDS use: daily, nondaily, or no use.

**Main Outcomes and Measures:**

The primary outcomes were 12 or more months’ abstinence from cigarette smoking and 12 or more months’ abstinence from both cigarette and ENDS use, ascertained at wave 6. Propensity score matching was used to control confounding on 14 potential confounders, including interest in quitting, income, age, education, nondaily smoking, and presence of a smoke-free home.

**Results:**

A total of 6013 smokers were included in the sample (3634 aged ≥35 years [weighted percentage, 65.2%]; 3182 female [weighted percentage, 46.5%]). Among smokers who vaped daily (228 individuals), an estimated 20.9% (95% CI, 15.0% to 26.8%) were abstinent from cigarette smoking at follow-up, compared with 14.3% abstinence (95% CI, 13.0% to 15.5%) among smokers who did not vape (5070 individuals) and 12.6% abstinence (95% CI, 9.8% to 15.4%) among smokers who vaped nondaily (715 individuals). Compared with similar propensity score–matched smokers who did not vape, smoking cessation was 4.1 percentage points lower among those who vaped daily (95% CI, −11.9 to 3.6 percentage points; *P* = .30), a nonsignificant difference. Smoking cessation was 5.3 percentage points lower among those who vaped nondaily (95% CI, −9.1 to −1.5 percentage points; *P* = .01) compared with similar propensity score–matched controls. Considering abstinence from both smoking and vaping, compared with matched controls, smokers who vaped daily had lower abstinence at follow-up by 14.7 percentage points (95% CI, −20.2 to −9.2 percentage points; *P* < .001), and those who vaped nondaily had lower abstinence by 7.2 percentage points (95% CI, −10.7 to −3.8 percentage points; *P* < .001).

**Conclusions and Relevance:**

In this representative cohort study of US smokers who used ENDS, neither daily nor nondaily vaping was associated with increased smoking cessation, and each was associated with reduced tobacco abstinence, suggesting that careful adjustment of confounding is critical in studies of ENDS and smoking cessation.

## Key Points

**Question:**

Is daily or nondaily vaping associated with increased smoking cessation after 4 years of follow-up?

**Findings:**

In this cohort study of 6013 cigarette smokers in the US, compared with matched controls who did not vape, smoking cessation rates were significantly lower (5.3 percentage points) among those who vaped nondaily; there was no statistically significant difference for those who vaped daily. Abstinence from both vaping and smoking was significantly lower (14.7 percentage points) among those who vaped daily.

**Meaning:**

In this cohort study, neither daily nor nondaily vaping among US smokers was associated with increased smoking cessation; however, each was associated with reduced tobacco abstinence, suggesting that vaping may prolong both smoking and nicotine dependence among US smokers.

## Teaser

This cohort study examines associations of vaping with long-term tobacco cessation among US cigarette smokers who used electronic nicotine delivery systems.

## Introduction

From 2014 to 2016, US smokers trying to quit were more likely to report using an e-cigarette than a US Food and Drug Administration–approved cessation aid to help with the quit attempt.^1^ E-cigarettes include all electronic nicotine delivery systems (ENDS).^2^ By 2020, 68% of current US smokers believed that ENDS use might help with smoking cessation.^3^ However, there is no corresponding consensus in the scientific literature. While a recent Cochrane review^4^ of randomized trials concluded that e-cigarettes increase smoking cessation more than nicotine replacement therapy, a meta-analysis^5^ that also included observational studies noted substantial heterogeneity and concluded that e-cigarette use is not generally associated with smoking cessation, although daily vaping might be helpful. A 2021 US Preventive Services Task Force review^6^ concluded that evidence of benefits from vaping was both limited and inconsistent.

There may be several reasons for such heterogeneity of results. Commercially available e-cigarettes have changed dramatically over time, notably with the widespread adoption of e-cigarettes with high concentrations of nicotine in the US in 2017.^7^ It is possible that more potent e-cigarettes may be more effective for smoking cessation.^8^ Different studies have used different outcomes, with some using short-term abstinence (ie, <6 months) as the outcome measure^9,10^ and others using at least 6 months’ abstinence.^11,12^ Differences in inclusion criteria for the comparison group, particularly on baseline level of interest in quitting cigarettes, may also potentially confound results.^13^

In this study, we used data from all adult current smokers from wave 4 (2017) of the nationally representative US Population Assessment of Tobacco and Health (PATH) cohort^14,15,16^ to investigate the association of daily and nondaily vaping in 2017 with 12 or more months’ abstinence at 4-year follow-up in 2021 (wave 6). We considered 2 abstinence outcomes: 12 or more months’ abstinence from cigarette smoking and 12 or more months’ abstinence from both smoking and vaping. We used propensity score matching because of its relative transparency and ease of interpretation, and, following statistical recommendations,^17^ matched on a comprehensive list of baseline potential confounders, while being careful to exclude potential mediating variables. We used the matching results to demonstrate the degree of potential confounding in unadjusted analyses from this observational study. As a complementary analysis, we used multivariable logistic regression to investigate these same comparisons.

## Methods

### Data Source

The PATH Study obtained written participant informed consent and was overseen by the Westat Institutional Review Board. This cohort study was a secondary data analysis that used deidentified data and had no contact with human participants, which met the criteria for exemption from further human participants’ protection review by the University of California, San Diego institutional review board. The reporting of the study follows the Strengthening the Reporting of Observational Studies in Epidemiology (STROBE) reporting guideline. The PATH cohort study sampled from a representative address-based list of US households, oversampling tobacco users, young adults aged 18 to 24 years, and African American individuals; respondents are regularly reinterviewed using computer-assisted methods.^14^ At wave 4, 27 757 adults from wave 1 were reinterviewed from December 1, 2016, through January 3, 2018, along with an additional randomly selected replenishment sample (6065 individuals).^15^ This wave 4 sample was reinterviewed in 2019 (wave 5) and 2021 (wave 6; March to September). Household screener response rates were 54% for the initial sample and 52.8% for the replenishment sample. Adult survey response rates were greater than 60% for all surveys. We use the PATH restricted use data files in this study.^16^

### Current Tobacco Use Status at Wave 4

For each PATH survey, lifetime cigarette smokers (ie, those who have smoked 100 or more cigarettes) were asked if they “currently smoke every day, some days, or not at all.” Those responding every day or some days were categorized as current established cigarette smokers. Respondents were also asked, “Do you now use electronic nicotine products every day, some days, or not at all?”

### Study Outcomes

Study outcomes were defined as abstinence for at least 12 months from (1) cigarette smoking and (2) cigarette and/or ENDS use at wave 6. Respondents were asked if they had ever used any electronic nicotine products, if they currently smoke or use electronic nicotine products every day or some days, and if they had smoked cigarettes or used an electronic nicotine product within the past 12 months.

### Potential Confounders at Wave 4

As in prior studies,^18,19^ potential confounders associated with smoking behavior included sociodemographic variables (age, sex, education, and income), self-reported race and ethnicity chosen from among survey options, cigarette smoking status (daily or nondaily), e-cigarette vaping status (daily, nondaily, or no use), interest in quitting cigarettes, having made a quit attempt in the past year, the perceived harm of cigarettes, insurance status, the presence of a smoke-free home, the existence of externalizing and/or internalizing mental health symptoms, and diagnosis of a smoking-related disease. We categorized race and ethnicity into White and other, with the latter including African-American or Black, Asian, Hispanic, and multiracial participants; race and ethnicity were included because smoking behaviors vary across different racial and ethnic groups.^20^ Measurement details are in the eAppendix in Supplement 1.

### Statistical Analysis

Analyses were conducted in R version 4.4.2 (R Project for Statistical Computing) from June 2023 to June 2024. Population estimates used the wave 6 all-waves longitudinal weights, which adjust for baseline nonresponse and longitudinal study dropout; inference was conducted using replicate weights with balanced repeated replication and Fay adjustment.^21^ Statistical significance was assessed at the 5% level (ie, a 2-tailed *P* < .05). Differences among weighted proportions were assessed by Pearson χ^2^ test (R package, survey). Prior to conducting the analysis, we used multivariate imputation by chained equations (R package, mice) to impute missing data in covariates.

Differences in cessation rates between ENDS users and propensity score–matched controls who did not vape were used to estimate the average causal effect of vaping at baseline on cigarette smoking abstinence at follow-up among the population of US cigarette smokers who use ENDS for 3 exposure groups: daily ENDS use, nondaily ENDS use, and any ENDS use (daily or nondaily). We estimated each of the 3 propensity scores separately using logistic regression with the corresponding exposure outcome, the subsample of exposed and control respondents, and the complete list of potential baseline confounders. We then matched each participant in the exposed group with 2 controls with a similar propensity score using 2:1 nearest-neighbor matching without replacement and a caliper of 0.1 (R package, MatchIt); the difference in abstinence rates between the exposed group and the matched controls (R package, survey) is the estimated average causal effect of e-cigarette use in the exposed group.^22^ Conceptually, for each ENDS user (the index case) the average outcome of the pair of matched control nonusers is used to impute the cessation outcome that would have occurred if the index case had not used ENDS. Hence, for these comparisons we used the survey weights of the index case.^23^ To assess the quality of the matching, we utilized a Love plot to visualize the absolute standardized mean difference between cases and controls for each potential confounder. As a sensitivity analysis, multivariable logistic regression with survey weights (R package, survey) was used to assess the associations of abstinence outcomes with baseline covariates.

## Results

### Baseline Characteristics and Vaping Status

At wave 4 (2017), there were 9915 current established cigarette smokers, of whom 6013 (3634 aged 35 years or older [weighted percentage, 65.2%]; 3182 female [weighted percentage, 46.5%]; 3779 White [weighted percentage, 68.0%]; 2234 other race [weighted percentage, 32.0%]) completed wave 5 and wave 6, comprising the study sample (Table 1). An estimated 14.4% (95% CI, 13.4%-15.0%) of current adult US smokers also vaped e-cigarettes in 2017, with 228 (weighted percentage, 3.9%) vaping daily and 715 (weighted percentage, 10.6%) vaping nondaily. At baseline, daily vaping was associated with nondaily vs daily smoking status (χ^2^_1_ = 158.25; *P* < .001) and with having made a cigarette quit attempt in the past year (χ^2^_2_ = 38.68; *P* < .001). Daily vaping was also associated with age (χ^2^_1_ = 23.74; *P* < .001), race and ethnicity (χ^2^_1_ = 13.49; *P* = .002), income (χ^2^_2_ = 14.67; *P* = .01), perceived harmfulness of cigarettes (χ^2^_1_ = 12.47; *P* = .001), moderate or high scores on externalizing mental health symptoms (χ^2^_2_ = 39.26; *P* < .001), and high scores on internalizing mental health symptoms (χ^2^_2_ = 15.31; *P* = .005). Similar associations were generally observed when examining the subsample of smokers who vaped nondaily, but the magnitude of the differences were less marked.

**Table 1.** Vaping Among US Smokers by Baseline Characteristics, 2017^a^

Baseline characteristics	Sample size, No. (weighted %)	Weighted % (95% CI)^b^	
		Daily vaping	Nondaily vaping
Overall	6013 (100)	3.9 (3.3-5.0)	10.6 (9.7-11.0)
Age, y			
<35	2379 (34.8)	5.5 (4.4-6.6)	14.8 (13.3-16.4)
≥35	3634 (65.2)	3.0 (2.3-3.7)	8.3 (7.2-9.4)
Sex			
Male	2831 (53.5)	4.0 (3.2-4.9)	10.6 (9.2-11.9)
Female	3182 (46.5)	3.7 (2.8-4.5)	10.6 (9.5-11.7)
Educational level			
<High School	1712 (26.9)	3.0 (2.0-4.0)	10.1 (8.5-11.8)
High school graduate	1524 (30.1)	3.8 (2.5-5.0)	9.5 (8.0-10.9)
Some college or more	2777 (43.1)	4.5 (3.5-5.5)	11.6 (10.4-12.7)
Race and ethnicity^c^			
Non-Hispanic White	3779 (68.0)	4.5 (3.7-5.3)	11.2 (10.0-12.3)
Other	2234 (32.0)	2.5 (1.7-3.4)	9.3 (7.9-10.6)
Income, $			
<35 000	3607 (56.3)	3.0 (2.3-3.8)	11.0 (9.8-12.2)
≥35 000	2148 (39.0)	5.0 (3.9-6.2)	10.3 (8.7-11.9)
Missing	258 (4.7)	4.1 (0.8-7.4)	7.7 (4.2-11.3)
Cigarette smoking status			
Daily	4602 (76.2)	2.1 (1.7-2.6)	10.1 (9.1-11.0)
Nondaily	1411 (23.8)	9.5 (7.7-11.3)	12.2 (10.4-13.9)
No past-year quit attempt and low interest in quitting	2791 (47.2)	2.6 (1.7-3.4)	10.0 (8.7-11.3)
No past-year quit attempt and high interest in quitting	1146 (19.8)	3.4 (2.3-4.5)	7.7 (6.1-9.3)
Past-year quit attempt	2076 (33.0)	6.0 (4.7-7.3)	13.1 (11.5-14.7)
Smoke-free home^d^			
No	2707 (43.7)	3.2 (2.4-4.0)	11.2 (9.8-12.5)
Yes	3273 (56.3)	4.4 (3.5-5.4)	10.0 (8.8-11.3)
Perceived harmfulness of cigarettes^d^			
Low	425 (6.7)	7.2 (4.4-9.9)	13.9 (10.6-17.2)
Moderate to high	5577 (93.3)	3.6 (3.0-4.3)	10.3 (9.4-11.3)
Health insurance status			
None	1267 (21.3)	4.3 (2.8-5.8)	11.1 (9.3-13.0)
Yes	4746 (78.7)	3.8 (3.1-4.4)	10.4 (9.4-11.4)
Externalizing mental health symptoms			
Low	4149 (70.5)	2.9 (2.2-3.5)	9.2 (8.2-10.3)
Moderate	1531 (24.7)	6.2 (4.6-7.7)	13.3 (11.3-15.3)
High	333 (4.8)	6.8 (3.8-9.7)	16.1 (12.2-20.1)
Internalizing mental health symptoms			
Low	2949 (51.5)	3.3 (2.5-4.0)	8.9 (7.7-10.0)
Moderate	1435 (23.5)	3.4 (2.3-4.5)	11.1 (9.4-12.7)
High	1629 (25.0)	5.6 (4.0-7.1)	13.5 (11.4-15.7)
Existence of smoking-related disease			
No	5604 (94.5)	3.9 (3.3-4.6)	10.2 (9.3-11.1)
Yes	409 (5.5)	2.6 (1.0-4.3)	16.9 (12.3-21.4)

^a^ Data are from wave 4 (2017) of the Population Assessment of Tobacco and Health (PATH) study.^14,15,16^

^b^ Weighted US population estimate using wave 6 (cohort 4) all-wave longitudinal weights.

^c^ For race and ethnicity, groups categorized as other were African American or Black, Asian, Hispanic, and multiracial.

^d^ Missing values that are less than 1% of the overall sample of 6013 were excluded from the table.

### Population Rates of Abstinence at Follow-Up

The unadjusted population rate of 12 or more months’ abstinence from cigarettes in 2021 was estimated among smokers grouped by baseline ENDS use (Table 2). Among US smokers who vaped daily in 2017, an estimated 20.9% (95% CI, 15.0%-26.8%) were abstinent from cigarette smoking for 12 or more months in 2021, a rate more than 6 percentage points higher (95% CI, 0.5-12.8 percentage points) than the cessation rate among all US smokers who did not vape at baseline (weighted percentage, 14.3%; 95% CI, 13.0%-15.5%). Thus, in an analysis not adjusted for potential confounders, daily ENDS use was associated with increased smoking cessation. The unadjusted cessation rate was 1.7 percentage points lower among nondaily ENDS users (weighted percentage, 12.6%; 95% CI, 9.8%-15.4%) compared with those who did not use ENDS (weighted percentage, 14.3%; 95% CI, 13.0%-15.5%; χ^2^_2_ = 9.72; *P* = .02). When considering 12 or more months’ abstinence from both cigarettes and ENDS in 2021, the abstinence rate at baseline was 4.6 percentage points lower for both daily (weighted percentage, 7.1%; 95% CI, 3.2%-11.0%) and nondaily vapers (weighted percentage, 7.1%; 95% CI, 4.9%-9.3%) compared with those who did not vape at baseline (weighted percentage, 11.7%; 95% CI, 10.5-12.8%; χ^2^_2_ = 15.87; *P* = .002).

**Table 2.** Abstinence Outcomes Among US Smokers in 2021 by ENDS Use Status in 2017^a^

Status	ENDS use in 2017 based on wave 4 baseline frequency of use, No. (weighted %)	≥12 Months’ abstinence in 2021 (wave 6), weighted % (95% CI)^b^	
		Abstinence from cigarettes	Abstinence from both cigarettes and ENDS
No use	5070 (85.6)	14.3 (13.0-15.5)	11.7 (10.5-12.8)
Nondaily vaping	715 (10.6)	12.6 (9.8-15.4)	7.1 (4.9-9.3)
Daily vaping	228 (3.9)	20.9 (15.0-26.8)	7.1 (3.2-11.0)

Abbreviation: ENDS, electronic nicotine delivery systems.

^a^ Data are from wave 4 (2017) and wave 6 (2021) of the Population Assessment of Tobacco and Health (PATH) Study.^14,15,16^

^b^ Weighted US population estimate using wave 6 (cohort 4) all-wave longitudinal weights. For unadjusted abstinence outcomes by study covariates, see eTable 3 in Supplement 1.

### Propensity Score Matching

We considered the 3 exposure groups (daily vaping, nondaily vaping, and any vaping [daily or nondaily]) separately. Each propensity score was estimated from a logistic regression using baseline covariates and the respective combined exposure and control (nonvaping) group (eTable 1 in Supplement 1). We then used the estimated propensity score to match each exposure-group smoker (ENDS user) with 2 similar control smokers who did not vape. Nearly all ENDS users were successfully matched (226 of 228 daily ENDS users; 712 of 715 nondaily ENDS users; 940 of 943 any ENDS users).

**Figure 1.**
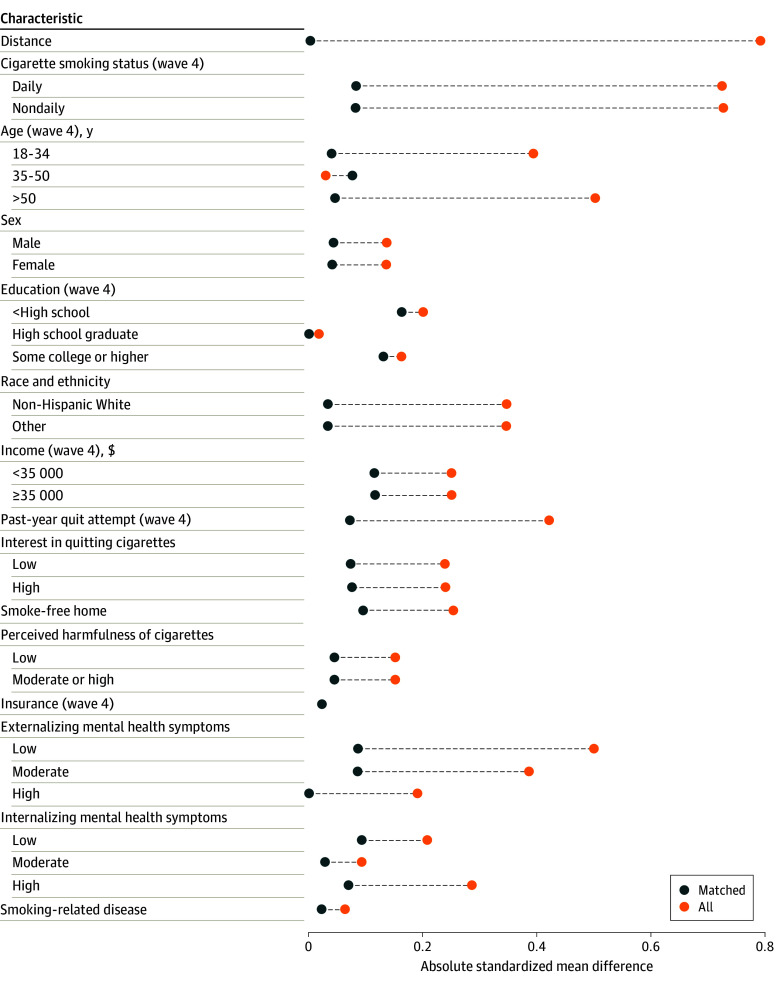
Absolute Standardized Mean Differences (Love Plot) for Potential Confounder Variables Comparing Current Smokers Who Vaped Daily vs Those Who Did Not Vape in 2017 Orange circles are differences in the full sample before matching and blue circles are differences in the 2:1 matched subsample. Distance is defined as the standardized mean difference between the groups in the estimated propensity score. Data are from wave 4 (2017) of the Population Assessment of Tobacco and Health (PATH) study.^14,15,16^

Considering the propensity score model for daily vaping (eTable 1 in Supplement 1), the adjusted odds of daily vaping were associated at baseline with the following factors: nondaily smoking (adjusted odds ratio [aOR], 4.33; 95% CI, 3.22-5.84), presence of a past-year quit attempt (aOR, 1.81; 1.34-2.46), interest in quitting smoking (aOR, 1.39; 95% CI, 1.02-1.89), age 50 years or older (aOR, 0.51; 95% CI, 0.33-0.75), race and ethnicity (aOR, 0.39; 95% CI, 0.28-0.54), moderate (aOR, 2.31; 95% CI, 1.63-3.27) and high (aOR, 2.97; 95% CI, 1.69-5.09) externalizing mental health symptoms, and perceived harmfulness of cigarettes (aOR, 0.37; 95% CI, 0.23-0.60). The imbalance in these covariates before matching is apparent in the Love plot (Figure 1). After matching, most cases and controls were very well balanced on all covariates (standardized mean difference <0.1); education and income were also balanced with standardized mean differences less than 0.2 (Figure 1).

When considering nondaily vaping, similar baseline associations were generally observed, but differences were less marked (eTable 1 in Supplement 1). In the propensity score model, covariate patterns were largely similar to the model for daily vaping, but effect sizes were smaller. Importantly, however, interest in quitting was not associated with nondaily vaping (aOR, 0.97; 95% CI, 0.81-1.16). Also, smokers with an existing smoking-related disease were more likely to report nondaily vaping (aOR, 1.62; 95% CI, 1.21-2.14), an association that was not observed with daily vaping. After matching, all covariates had standardized mean differences less than 0.1, indicating excellent balance (eFigure 1 in Supplement 1). Results for any vaping were similar to the results for nondaily vaping (eTable 1 and eFigure 2 in Supplement 1).

### Matched Analysis of Vaping and Abstinence Outcomes Among Smokers Who Used ENDS

**Figure 2.**
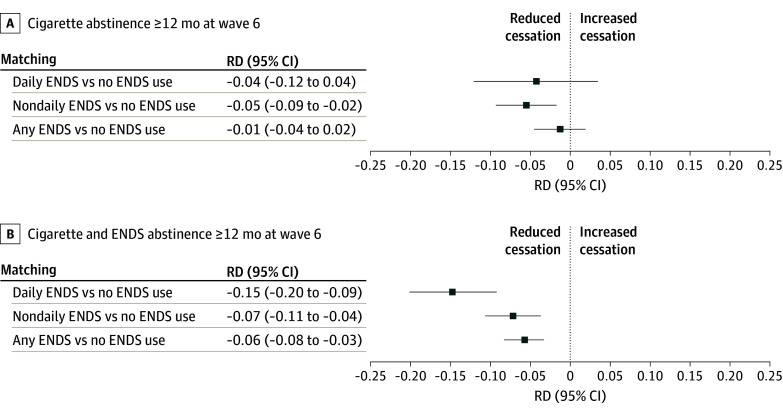
Estimated Differences in Abstinence Rates Between US Smokers Who Vape and Their Matched Controls Who Do Not Vape for Daily Vaping, Nondaily Vaping, and Any Vaping (Daily or Nondaily) Vaping assessed at baseline (2017) and abstinence assessed at follow-up (2021), for the outcomes of 12 or more months’ abstinence from cigarette smoking (A) and 12 or more months’ abstinence from both cigarette smoking and vaping (B). Risk difference (RD) indicates the difference in abstinence rates, smokers who vape minus controls. ENDS indicates electronic nicotine delivery systems.

To estimate the association of ENDS use with smoking cessation among those who did use ENDS, we estimated the population rate of 12 or more months’ cigarette abstinence in 2021 among the ENDS users and then compared the abstinence rate among their matched controls (Figure 2A). Smokers who vaped daily at baseline had a smoking cessation rate that was 4.1 percentage points lower than their matched controls (95% CI, −11.9 to 3.6 percentage points; *P* = .30), although this difference was not statistically significant. For nondaily vapers, vaping was associated with a reduction in smoking cessation of 5.3 percentage points (95% CI, −9.1 to −1.5 percentage points; *P* = .01). For the combined daily and nondaily vaping exposure group, the estimated effect size of ENDS use was much smaller and not statistically significant (percentage point difference, −1.13; 95% CI, −4.30 to 2.03; *P* = .48).

When considering the outcome of 12 or more months’ cessation of both cigarettes and e-cigarettes, daily vaping among US smokers who vaped was associated with a reduction in smoking cessation of 14.7 percentage points (95% CI, −20.2 to −9.2 percentage points; *P* < .001) (Figure 2B). Nondaily vaping among US smokers who vaped was associated with a reduction in cessation of smoking and vaping of 7.2 percentage points (95% CI, −10.7 to −3.8 percentage points; *P* < .001) and any vaping among US smokers was associated with a reduction in cessation of smoking and vaping of 5.77 percentage points (95% CI, −8.24 to −3.31 percentage points; *P* < .001).

### Sensitivity Analyses

As a complementary analysis, we estimated the association of daily and nondaily vaping with abstinence outcomes using 2 weighted multivariable logistic regression models on the whole sample (Table 3 and eTable 2 in Supplement 1). The unadjusted estimates of covariates are given in eTable 3 in Supplement 1. Considering 12 or more months’ abstinence from cigarettes in 2021, the daily vaping results were similar to the matching analysis; smokers who vaped daily did not have statistically different odds of abstinence compared with those who did not vape (aOR, 0.94; 95% CI, 0.62-1.41; *P* = .76). For smokers who vaped on a nondaily basis, the direction of the results was consistent with the matched analysis; however, this finding was not statistically significant in the logistic regression model (aOR, 0.77; 95% CI, 0.58-1.02; *P* = .06). (Table 3)

**Table 3.** Multivariable Logistic Regression Model of 12 or More Months’ Abstinence From Cigarettes^a^

Baseline characteristics (2017)	Abstinence, weighted % (95% CI)^b^	Cigarette abstinence in 2021	
		aOR (95% CI)	*P* value
E-cigarette vaping status			
No use	14.3 (13.0-15.5)	1 [Reference]	NA
Nondaily use	12.6 (9.8-15.4)	0.77 (0.58-1.02)	.06
Daily use	20.9 (15.0-26.8)	0.94 (0.62-1.41)	.76
Cigarette smoking status			
Daily	9.7 (8.7-10.8)	1 [Reference]	NA
Nondaily	29.1 (26.3-31.9)	3.17 (2.55-3.95)	<.001
Quitting history and interest in quitting			
No past-year quit attempt and low quit interest	11.9 (10.2-13.7)	1 [Reference]	NA
No past-year quit attempt and high quit interest	14.7 (12.0-17.4)	1.46 (1.08-1.95)	.01
Past-year quit attempt	17.6 (15.9-19.4)	1.34 (1.08-1.68)	.01
Smoke-free home			
No	10.2 (8.8-11.6)	1 [Reference]	NA
Yes	17.7 (16.1-19.2)	1.36 (1.13-1.64)	.001
Age, y			
≥35	12.8 (11.4-14.2)	1 [Reference]	NA
<35	17.3 (15.7-18.9)	1.23 (1.02-1.49)	.03
Education			
<High school	11.4 (8.8-14.0)	1 [Reference]	NA
High school graduate	11.7 (9.5-13.9)	0.99 (0.73-1.36)	.97
At least some college	18.0 (16.4-19.7)	1.43 (1.09-1.88)	.01
Sex			
Male	15.0 (13.6-16.4)	1 [Reference]	NA
Female	13.6 (11.9-15.3)	0.85 (0.70-1.05)	.13
Race and ethnicity^c^			
Non-Hispanic White	14.1 (12.7-15.6)	1 [Reference]	NA
Other	14.8 (13.0-16.7)	0.90 (0.74-1.10)	.31
Income, $			
<35 000	12.5 (11.2-13.8)	1 [Reference]	NA
≥35 000	16.8 (14.9-18.6)	1.01 (0.82-1.24)	.94
Harmfulness of cigs			
Low	12.4 (8.9-15.9)	1 [Reference]	NA
Moderate to high	14.5 (13.3-15.7)	0.94 (0.66-1.34)	.75
Insurance status			
No	11.7 (10.0-13.4)	1 [Reference]	NA
Yes	15.1 (13.8-16.3)	1.19 (0.97-1.46)	.10
Externalizing mental health symptoms			
Low	14.4 (13.0-15.8)	1 [Reference]	NA
Moderate	14.6 (12.3-16.9)	0.95 (0.74-1.23)	.70
High	12.0 (8.2-15.9)	0.79 (0.51-1.21)	.27
Internalizing mental health symptoms			
Low	14.8 (13.4-16.1)	1 [Reference]	NA
Moderate	14.3 (11.5-17.1)	0.92 (0.71-1.18)	.48
High	13.5 (11.6-15.4)	0.95 (0.75-1.20)	.66
Existent smoking-related disease			
No	14.4 (13.2-15.5)	1 [Reference]	NA
Yes	14.1 (10.1-18.1)	1.06 (0.71-1.58)	.78

Abbreviations: aOR, adjusted odds ratio; NA, not applicable.

^a^ Data are from wave 4 (2017) and wave 6 (2021) of the Population Assessment of Tobacco and Health (PATH) study.^14,15,16^

^b^ Weighted US population estimate of 12 or more months’ abstinence from cigarettes using the wave 6 cohort 4 all-wave longitudinal weights.

^c^ For race and ethnicity, groups categorized as other were African American or Black, Asian, Hispanic, and multiracial.

Considering 12 or more months’ abstinence from both cigarettes and ENDS at 2021 follow-up, smokers who vaped daily had lower odds of abstinence for both products compared with smokers who did not vape (aOR, 0.36; 95% CI, 0.19-0.69; *P* = .002). The same was true for smokers who vaped nondaily (aOR, 0.54; 95% CI, 0.37-0.78; *P* = .001) (eTable 2 in Supplement 1).

## Discussion

In this representative cohort study, as in prior studies, unadjusted abstinence rates suggested that daily e-cigarette vaping was associated with increased long-term smoking cessation.^9,19,24^ However, once confounders were adjusted for, daily vaping at baseline was not associated with increased cigarette abstinence at follow-up as compared with similar smokers who did not vape. In a propensity score–matched analysis, there was a nonsignificant difference of 4.1 percentage points (95% CI, −11.9 to 3.6 percentage points; *P* = .30) among those who vaped daily and a significant reduction in smoking cessation of 5.3 percentage points (95% CI, −9.1 to −1.5 percentage points; *P* = .01) among those who vaped nondaily. Logistic regression models applied to the US population of smokers gave qualitatively similar results. Considering the outcome of 12 or more months’ abstinence from both smoking and vaping, results of both analytic approaches were similar but estimated effect sizes were larger. In the propensity score–matched analysis, daily vaping reduced tobacco abstinence rates by 14.7 percentage points (95% CI, −20.2 to −9.2 percentage points; *P* < .001) compared with matched controls, and nondaily vaping by 7.2 percentage points (95% CI, −10.7 to −3.8 percentage points; *P* < .001), suggesting that many dual smokers and ENDS users remain dependent on e-cigarettes even if they manage to quit smoking.^25,26^

Our analyses confirmed that a comparison of unadjusted smoking cessation rates between ENDS users (daily or nondaily) and nonusers in the US population is confounded, even in prospective analysis.^13^ The degree of confounding was such that the estimated effect size of ENDS use changed from seemingly beneficial in unadjusted analysis to deleterious once accounting for confounding. Smokers with baseline characteristics associated with future smoking cessation,^18,19^ such as recent quit attempts, interest in quitting, or nondaily smoking status, were much more likely to use ENDS daily. Additional identified confounders included age, race and ethnicity, mental health symptoms, presence of a smoke free home, and perceived harmfulness of cigarettes.

There are several related analyses of the PATH data, which largely address the older style ENDS, which were commercially available prior to 2017. Chen et al^19^ reported that 12 or more months’ cigarette abstinence among those who used ENDS to help with a quit attempt was substantially and significantly lower than for similar smokers who made a quit attempt without using an aid. Pierce et al^27^ considered cigarette smokers at baseline who had quit cigarettes 1 year later; those who switched to e-cigarettes (whether daily or not) had similar rates of relapse to cigarette smoking over the following year as those who were tobacco-free at the first follow-up. Kasza, et al^10^ studied daily smokers who used ENDS between 2014 and 2019 and concluded that using ENDS daily (vs nondaily) was associated with greater cessation success. Each of these studies is consistent with our results. Harlow et al^28^ selected wave-1 PATH respondents who smoked cigarettes but did not vape and reported 12 or more months’ cigarette abstinence at wave 4. Using marginal structural models and inverse probability weighting, they estimated that the less than 2% of the sample who vaped daily at both wave 2 and wave 3 had double the cessation rate of others. A concern was that adjustment for covariates did not result in substantial differences between adjusted and unadjusted estimates, suggesting inadequate control of confounding.

### Strengths and Limitations

A major strength is the use of the state-of-the-art US PATH longitudinal cohort that provides population-based estimates of tobacco use changes over time. Use of such high-quality observational data increases the generalizability of reported results to the US population. To address self-selection bias, we matched each e-cigarette user to a nonuser on a comprehensive list of baseline variables that are associated with both e-cigarette use and smoking cessation and effectively controlled for confounding. Nevertheless, this study has limitations. Undoubtedly, there was still some unmeasured residual confounding between product user groups. Following statistical recommendations, we did not include mediator variables (such as cigarette dependence measures) in the set of control variables. We assumed that most cigarette dependence variables are intermediate variables on the causal pathway, likely to be affected by ENDS use. We chose to control for daily and nondaily smoking status, assuming that most daily smokers did not switch to nondaily status because of ENDS use. These assumptions, however, should be investigated in future work.

## Conclusions

In this cohort study of a representative sample of US adult smokers, undertaken when high-nicotine e-cigarettes were readily available on the market, e-cigarette use was not associated with increased future smoking cessation. Although unadjusted cessation rates in the population appeared to favor the daily use of e-cigarettes, when major confounders were controlled for, the estimated effect size indicated a decrease in future smoking cessation among smokers who vape, whether they vaped daily or nondaily. Furthermore, e-cigarette use was associated with a large and significant decrease in future abstinence from tobacco use (cigarettes or ENDS). These nationally representative data suggest that vaping prolongs both smoking and nicotine dependence among US smokers.^26^

## Notes

Supplement 1. **eAppendix.** Measurement Detail for Study Covariates **eTable 1.** Logistic Regression Propensity Score Models of ENDS Use at Baseline **eFigure 1.** Absolute Standardized Mean Differences (Love Plot) for Potential Confounder Variables Between Current Smokers Who Vaped on a Non-Daily Basis Versus Those Who Did Not Vape **eFigure 2.** Absolute Standardized Mean Differences (Love Plot) for Potential Confounder Variables Between Current Smokers Who Vaped (Either Daily or Non-Daily) Versus Those Who Did Not Vape **eTable 2.** Logistic Regression Modeling 12+ Month Abstinence From Both Cigarettes and E-Cigarettes at Wave 6 (2021) by Baseline Covariates **eTable 3.** 12+ Month Abstinence at Wave 6 (2021) by Baseline (W4, 2017) Characteristics

Supplement 2.
Data Sharing Statement
