# Supplementary material for: Daily or Nondaily Vaping and Smoking Cessation Among Smokers
Source: JAMA Netw Open. 2025 Mar 5;8(3):e250089. doi: 10.1001/jamanetworkopen.2025.0089 (PMC11883493; doi:10.1001/jamanetworkopen.2025.0089)
Supplement: Supplement 1. — eAppendix. Measurement Detail for Study Covariates eTable 1. Logistic Regression Propensity Score Models of ENDS Use at Baseline eFigure 1. Absolute Standardized Mean Differences (Love Plot) for Potential Confounder Variables Between Current Smokers Who Vaped on a Non-Daily Basis Versus Those Who Did Not Vape eFigure 2. Absolute Standardized Mean Differences (Love Plot) for Potential Confounder Variables Between Current Smokers Who Vaped (Either Daily or Non-Daily) Versus Those Who Did Not Vape eTable 2. Logistic Regression Modeling 12+ Month Abstinence From Both Cigarettes and E-Cigarettes at Wave 6 (2021) by Baseline Covariates eTable 3. 12+ Month Abstinence at Wave 6 (2021) by Baseline (W4, 2017) Characteristics [file jamanetwopen-e250089-s001.pdf]

Supplemental Online Content

Quach NE, Pierce JP, Chen J, et al. Daily or nondaily vaping and smoking cessation among smokers. *JAMA Netw. Open*. 2024;8(3):e250089. doi:10.1001/jamanetworkopen.2025.0089

eAppendix. Measurement Detail for Study Covariates
eTable 1. Logistic Regression Propensity Score Models of ENDS Use at Baseline
eFigure 1. Absolute Standardized Mean Differences (Love Plot) for Potential Confounder Variables Between Current Smokers Who Vaped on a Non-Daily Basis Versus Those Who Did Not Vape
eFigure 2. Absolute Standardized Mean Differences (Love Plot) for Potential Confounder Variables Between Current Smokers Who Vaped (Either Daily or Non-Daily) Versus Those Who Did Not Vape
eTable 2. Logistic Regression Modeling 12+ Month Abstinence From Both Cigarettes and E-Cigarettes at Wave 6 (2021) by Baseline Covariates
eTable 3. 12+ Month Abstinence at Wave 6 (2021) by Baseline (W4, 2017) Characteristics

This supplemental material has been provided by the authors to give readers additional information about their work.

© 2025 Quach NE et al. *JAMA Network Open*.

eAppendix. Measurement Detail for Study Covariates

Socio-demographics: Use standard derived variables for age, sex, education, race, ethnicity, and income (R04R_A_AGE, R04R_A_SEX_IMP, R04R_A_EDUC4_IMP, R04R_A_RACE_IMP, R04R_A_HISP_IMP, R04_AM0030). The quantitative variable age was categorized as "<35" years old and ">=35" years old to represent younger adults and older adults.

Cigarette smoking status at W4: Smoked cigarettes daily or non-daily at W4 (R04_AC1003).

E-cigarette vaping at W4: Used electronic nicotine products daily, non-daily, or not at all at W4 (R04_AV1003).

Past year quit attempt: Respondents were asked if in the past 12 months, have they tried to quit smoking completely (R04_AN0105) and the number of times they tried to quit smoking completely (R04_AN0115). If the respondent answered yes to R04_AN0105 and had a response greater than zero to R04_AN0115, the respondent was counted as making a past year quit attempt.

Interest in quitting cigarettes: On a scale of 1-10 where 1=Not at all interested and 10=Extremely interested (R04_AN0230). A median split was used on the variable R04_AN0230 so that the interested in quitting cigarettes variable has categories "Low" and "High".

Quitting history and interest in quitting: Derived using the past year quit attempt and interested in quitting cigarettes variables above. If the respondent make a past year quit attempt, the value was assigned as any past year quit attempt. If the respondent did not make a past year quit attempt and had a high interest in quitting cigarettes, the value was assigned as no past year quit attempt, high quit interest. If the respondent did not make a past year quit attempt and had a low interest in quitting cigarettes, the value was assigned as no past year quit attempt, low quit interest.

Smoke-free home: Using the variable R04_AR1045, a statement that best describes rules about smoking a combustible tobacco product inside home. It is a 3-point scale: 1 (not allowed anywhere or anytime at all), 2 (allowed in some places or sometimes), and 3 (allowed anywhere or anytime at all). The variable R04_AR1045 was dichotomized to create a binary smoke-free home variable (yes/no).

Perceived harmfulness of cigarettes: Respondents were asked "How harmful do you think cigarettes are to health?" and could reply on a 5-point scale from 1 (not at all harmful) to 5 (extremely harmful) (R04_AC9050). Values 1 and 2 of the variable R04_AC9050 were categorized as "Low" and values 3, 4, and 5 of R04_AC9050 were categorized as "Moderate/High".

Insurance status: (R04_AM0026_01 to R04_AM0026_08) Respondents who reported currently being covered by at least one type of health insurance, including insurance purchased directly or through an employer or union, Medicare, Medicaid, VA, TRICARE or other military health care and Indian Health Insurance, were scored as having insurance coverage. Missing data on all of these variables were coded to "did not have insurance".

External mental health symptoms: Respondents were asked the last time they had experienced any of 7 externalizing mental health symptoms (e.g., had a hard time paying attention or listening to instructions at school, work or home, bullied or started physical fights) from the following variables: R04_AX0165, R04_AX0166, R04_AX0167, R04_AX0168, and R04_AX0169. The number of reports of experiencing such symptoms in the past month or the past 2-12 months was summed and coded into a 3-level severity indicator, with those reporting 0 or 1 symptom scored as Low, 2-3 symptoms scored as Moderate and 4 or more scored as High.

Internal mental health symptoms: Respondents were asked the last time they experienced any of 4 internalizing disorder symptoms (feeling very trapped, lonely, sad, blue, depressed, or hopeless about the future, feeling very anxious, nervous, tense, scared, panicked, or like something bad was going to happen, had sleep problems) as measured by the following variables: R04_AX0161, R04_AX0162, R04_AX0163, and R04_AX0164. The number of reports of experiencing such symptoms in the past month or the past 2-12 months was summed and coded into a 3-level severity indicator, with those reporting 0 or 1 symptom scored as Low, 2-3 symptoms scored as Moderate and 4 or more scored as High.

Existence of smoking-related disease: Respondents were asked if they had ever been told by a doctor or health professional that they had any of the listed diseases.
Group A: Heart Disease: High blood pressure (R04_AX0111_NB_01), High cholesterol (R04_AX0111_NB_02) Congestive heart failure (R04_AX0111_NB_03); a stroke (R04_AX0111_NB_04); A heart attack (R04_AX0111_NB_05); Some other heart condition (R04_AX0111_NB_06)
Group B: Respiratory Disease: COPD (R04_AX0119_NB_01); chronic bronchitis (R04_AX0119_NB_02); emphysema (R04_AX0119_NB_03); asthma (R04_AX0119_NB_04); some other lung or respiratory condition (R04_AX0119_NB_05)
Group C: Cancer: (R04_AX0144 _NB)

© 2025 Quach NE et al. *JAMA Network Open*.

eTable 1: Logistic Regression Propensity Score Models of ENDS use at Baseline (W4).

Model 1: daily vaping vs no vaping, Model 2: non-daily vaping vs no vaping; Model 3: any vaping (daily or non-daily) vs no vaping

			Propensity Score Model 1		Propensity Score Model 2		Propensity Score Model 3	
**Wave 4 Variables**		**n**	**AOR (95% CI)**	**P-value**	**AOR (95% CI)**	**P-value**	**AOR (95% CI)**	**P-value**
Cigarette Smoking Status	Daily	4602	[Reference]					
	Non-daily	1411	4.33 (3.22, 5.84)	<0.01	1.36 (1.13, 1.65)	<0.01	1.84 (1.56, 2.17)	<0.01
Age	18-34	2379	[Reference]					
	35-50	1739	0.84 (0.60, 1.16)	0.29	0.64 (0.53, 0.78)	<0.01	0.68 (0.57, 0.81)	<0.01
	50+	1895	0.51 (0.33, 0.75)	<0.01	0.43 (0.34, 0.53)	<0.01	0.44 (0.36, 0.54)	<0.01
Sex	Male	2831	[Reference]					
	Female	3182	0.78 (0.59, 1.04)	0.09	0.98 (0.83, 1.15)	0.80	0.94 (0.81, 1.09)	0.41
Education	< High school	1712	[Reference]					
	High school graduate	1524	1.19 (0.79, 1.79)	0.41	0.97 (0.78, 1.22)	0.82	1.02 (0.84, 1.26)	0.82
	Some college+	2777	1.04 (0.72, 1.53)	0.83	1.14 (0.93, 1.40)	0.22	1.12 (0.93, 1.35)	0.22
Race/ethnicity[a]	Non-Hispanic white	3779	[Reference]					
	Others	2234	0.39 (0.28, 0.54)	<0.01	0.73 (0.62, 0.87)	<0.01	0.64 (0.54, 0.75)	<0.01
Income (US$)	<35,000	3607	[Reference]					
	>= $35,000	2148	1.31 (0.96, 1.79)	0.09	1.01 (0.84, 1.21)	0.91	1.08 (0.92, 1.27)	0.35
Past year quit attempt	No	3937	[Reference]					
	Yes	2076	1.81 (1.34, 2.46)	<0.01	1.39 (1.17, 1.66)	<0.01	1.48 (1.27, 1.74)	<0.01
Interested in quitting cigarettes	Low	3508	[Reference]					
	High	2505	1.39 (1.02, 1.89)	0.04	0.97 (0.81, 1.16)	0.74	1.05 (0.89, 1.23)	0.56
Smoke-free home	No	2707	[Reference]					
	Yes	3273	0.99 (0.73, 1.36)	0.95	0.83 (0.70, 0.99)	0.04	0.86 (0.74, 1.00)	0.05
Perceived harmfulness of cigarettes	Low	425	[Reference]					
	Moderate/High	5577	0.37 (0.23, 0.60)	<0.01	0.54 (0.41, 0.71)	<0.01	0.50 (0.39, 0.65)	<0.01
Insurance status	No	1267	[Reference]					
	Yes	4746	0.87 (0.62, 1.25)	0.44	0.94 (0.77, 1.14)	0.52	0.92 (0.77, 1.11)	0.38
External mental health symptoms	Low	4149	[Reference]					
	Moderate	1531	2.31 (1.63, 3.27)	<0.01	1.30 (1.06, 1.60)	0.01	1.49 (1.24, 1.79)	<0.01
	High	333	2.97 (1.69, 5.09)	<0.01	1.45 (1.03, 2.02)	0.03	1.67 (1.23, 2.26)	<0.01
Internal mental health symptoms	Low	2949	[Reference]					
	Moderate	1435	0.78 (0.52, 1.16)	0.24	1.04 (0.84, 1.28)	0.75	0.98 (0.81, 1.19)	0.85
	High	1629	1.03 (0.70, 1.53)	0.87	1.14 (0.91, 1.43)	0.26	1.12 (0.91, 1.37)	0.27
Existence of smoking-related disease	No	5604	[Reference]					
	Yes	409	0.88 (0.45, 1.57)	0.69	1.62 (1.21, 2.14)	<0.01	1.46 (1.11, 1.90)	0.01

AOR: adjusted odds ratio

[a]For race/ethnicity, groups categorized as “Others” were Hispanic, African American/Black, Asian and multi-racial

© 2025 Quach NE et al. *JAMA Network Open*.

eFigure 1: Absolute standardized mean difference (Love plot) for potential confounder variables between current smokers who vaped on a non-daily basis versus those who did not vape. Open circles are differences before matching, solid circles are differences after matching. R04_AC1003, cigarette smoking status at Wave 4.

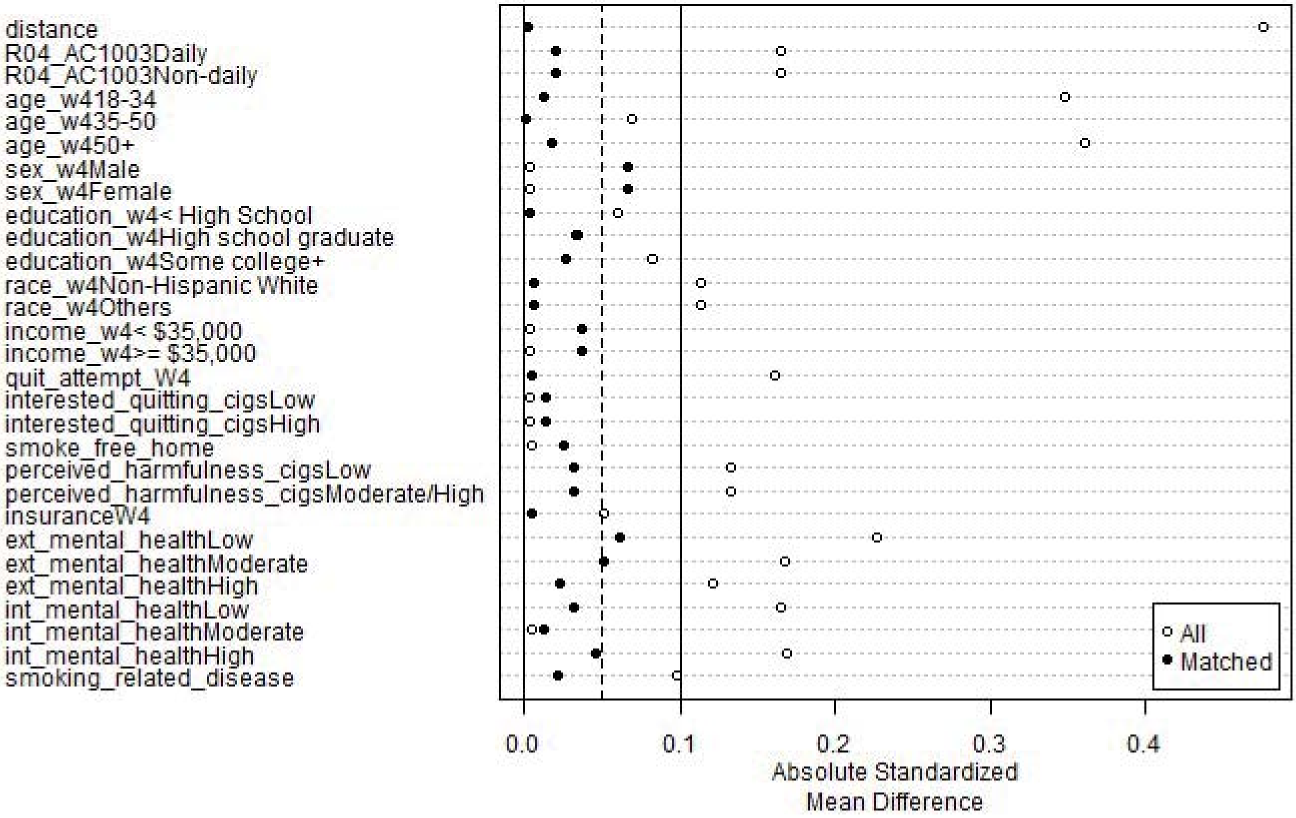

In the unmatched samples, the absolute standardized mean difference between study groups was unbalanced (>0.1) for daily/non-daily e-cigarette use, age, quit attempt, externalized mental health symptoms, internalized mental health symptoms, perceived harmfulness of cigarettes, and race-ethnicity. After matching, all covariates were well balanced.

© 2025 Quach NE et al. *JAMA Network Open*.

eFigure 2: Absolute standardized mean differences (Love plot) for potential confounder variables between current smokers who vaped (daily or non-daily) versus those who did not vape. Open circles are differences before matching, solid circles are differences after matching. R04_AC1003, cigarette smoking status at Wave 4.

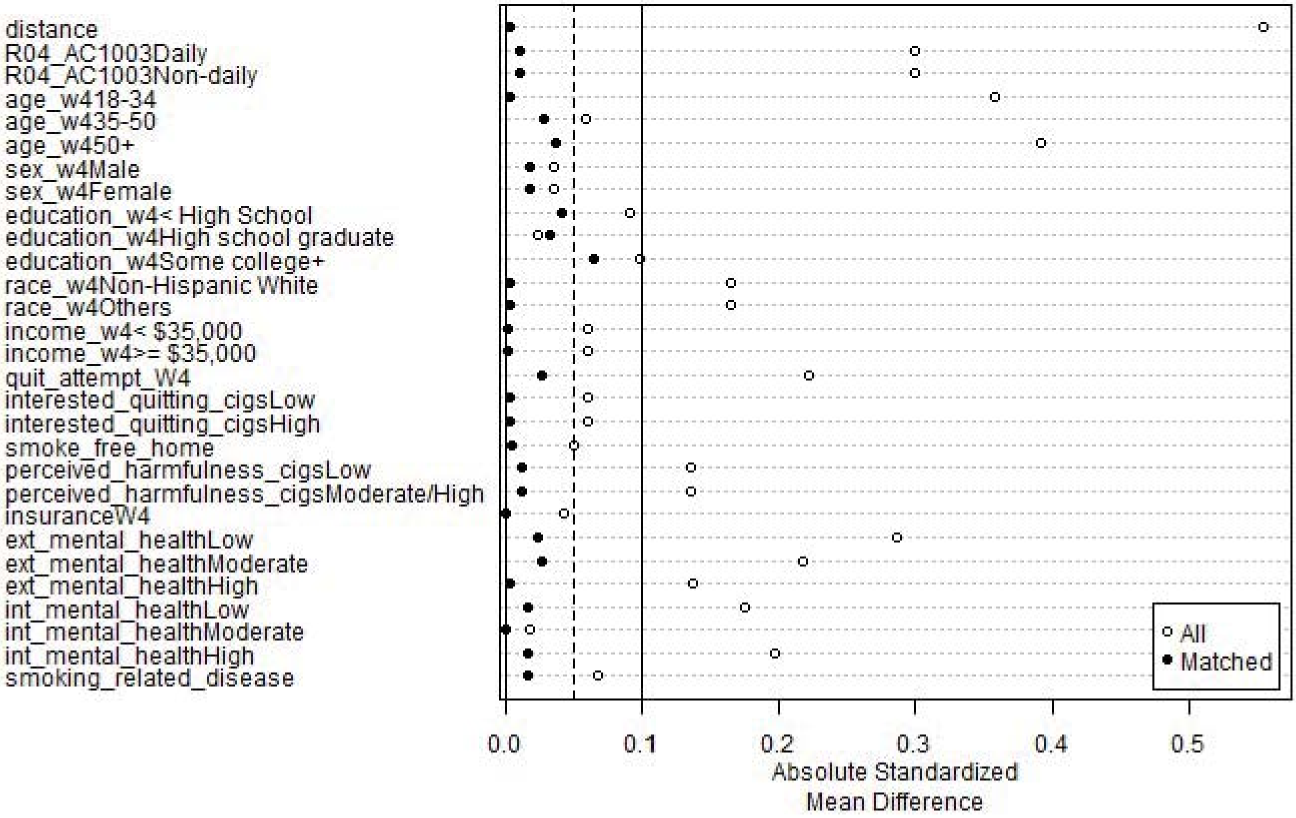

In the unmatched samples, the absolute standardized mean difference between study groups was unbalanced (>0.1) for daily/non-daily e-cigarette use, age, race-ethnicity, quit attempt, externalized mental health symptoms, internalized mental health symptoms, perceived harmfulness of cigarettes. After matching, all covariates were well balanced.

© 2025 Quach NE et al. *JAMA Network Open*.

eTable 2: Logistic Regression Modeling 12+ month Abstinence from both Cigarettes and E-cigarettes at Wave 6 (2021)

Baseline Characteristics (2017)		n	AOR	95% CI		P-value
ENDS status	No Use	5070	[Reference]			
	Daily	228	**0.36**	0.19	0.69	<0.01
	Non-daily	715	**0.54**	0.37	0.78	<0.01
Cigarette smoking status	Daily	4602	[Reference]			
	Non-daily	1411	**3.48**	2.78	4.35	<0.001
Age	>= 35	3634	[Reference]			
	< 35	2379	0.98	0.77	1.25	0.88
Sex	Male	2831	[Reference]			
	Female	3182	0.84	0.67	1.05	0.13
Education	< High school	1712	[Reference]			
	High school graduate	1524	0.83	0.58	1.19	0.31
	Some college+	2777	**1.41**	1.05	1.91	0.02
Race/ethnicity[a]	Non-Hispanic white	3779	[Reference]			
	Others	2234	1.05	0.84	1.30	0.68
Income (US$)	<35,000	3607	[Reference]			
	>=35,000	2148	1.01	0.80	1.28	0.93
Quitting history and interest in quitting	No past year quit attempt, low interest	2791	[Reference]			
	Any past year quit attempt	2076	**1.31**	1.04	1.65	0.02
	No past year quit attempt, high interest	1146	1.38	1.01	1.89	0.04
Smoke-free home	No	2707	[Reference]			
	Yes	3273	1.22	0.97	1.54	0.10
Perceived harmfulness of cigarettes	Low	425	[Reference]			
	Moderate/High	5577	1.16	0.77	1.75	0.46
Insurance status	No	1267	[Reference]			
	Yes	4746	1.27	0.99	1.63	0.06
External mental health symptoms	Low	4149	[Reference]			
	Moderate	1531	0.81	0.61	1.06	0.13
	High	333	0.74	0.48	1.13	0.16
Internal mental health symptoms	Low	2949	[Reference]			
	Moderate	1435	0.88	0.66	1.18	0.38
	High	1629	0.91	0.68	1.22	0.52
Existence of smoking-related disease	No	5604	[Reference]			
	Yes	409	0.99	0.62	1.59	0.98

AOR: adjusted odds ratio

[a]For race/ethnicity, groups categorized as "Others" were Hispanic, African American/Black, Asian, and multi-racial.

© 2025 Quach NE et al. *JAMA Network Open*.

eTable 3: 12+ month Abstinence at Wave 6 (2021) by Baseline (2017) Characteristics				
Baseline Variables		n	Abstinence from Cigarettes at W6	Abstinence from both Cigarettes and ENDS
			Wtd[a] (95% CI)	Wtd[a] (95% CI)
Overall		6013	14.3 (13.2, 16.0)	11.0 (10.0, 12.0)
ENDS status	No use	5070	14.3 (13.0, 15.5)	11.7 (10.5, 12.8)
	Daily	228	20.9 (15.0, 26.8)	7.1 (3.2, 11.0)
	Non-daily	715	12.6 (9.8, 15.4)	7.1 (4.9, 9.3)
Age	>= 35	3634	12.8 (11.4, 14.2)	10.8 (9.4, 12.2)
	<35	2379	17.3 (15.7, 18.9)	11.5 (10.1, 12.9)
Sex	Male	2831	15.0 (13.6, 16.4)	11.5 (10.3, 12.7)
	Female	3182	13.6 (11.9, 15.3)	10.4 (8.9, 12.0)
Education	< High School	1712	11.4 (8.8, 14.0)	9.3 (6.8, 11.8)
	High school graduate	1524	11.7 (9.5, 13.9)	8.2 (6.4, 10.0)
	Some college+	2777	18 (16.4, 19.7)	14.0 (12.5, 15.6)
Race/ethnicity[b]	Non-Hispanic white	3779	14.1 (12.7, 15.6)	10.3 (9.0, 11.5)
	Others	2234	14.8 (13.0, 16.7)	12.6 (10.7, 14.5)
Income (US$)	<35,000	3607	12.5 (11.2, 13.8)	9.6 (8.5, 10.7)
	>=35,000	2148	16.8 (14.9, 18.6)	12.9 (11.2, 14.6)
Cigarette smoking status	Daily	4602	9.7 (8.7, 10.8)	7.4 (6.6, 8.2)
	Non-daily	1411	29.1 (26.3, 31.9)	22.8 (20.0, 25.5)
Quitting history and interest in quitting	No past year quit attempt, low interest	2791	11.9 (10.2, 13.7)	9.3 (7.8, 10.8)
	No past year quit attempt, high interest	1146	14.7 (12.0, 17.4)	11.1 (8.7, 13.6)
	A past year quit attempt	2076	17.6 (15.9, 19.4)	13.4 (11.8, 15.0)
Smoke-free home	No	2707	10.2 (8.8, 11.6)	8.2 (7.0, 9.4)
	Yes	3273	17.7 (16.1, 19.2)	13.3 (11.8, 14.7)
Perceived harmfulness of cigarettes	Low	425	12.4 (8.9, 15.9)	7.5 (4.8, 10.1)
	Moderate/High	5577	14.5 (13.3, 15.7)	11.3 (10.2, 12.3)
Insurance status	No	1267	11.7 (10.0, 13.4)	8.4 (6.9, 9.8)
	Yes	4746	15.1 (13.8, 16.3)	11.7 (10.6, 12.9)
External mental health symptoms	Low	4149	14.4 (13.0, 15.8)	11.7 (10.5, 13.0)
	Moderate	1531	14.6 (12.3, 16.9)	9.6 (7.7, 11.4)
	High	333	12.0 (8.2, 15.9)	8.2 (5.0, 11.3)
Internal mental health symptoms	Low	2949	14.8 (13.4, 16.1)	11.9 (10.7, 13.0)
	Moderate	1435	14.3 (11.5, 17.1)	10.9 (8.3, 13.4)
	High	1629	13.5 (11.6, 15.4)	9.3 (7.5, 11.2)
Existence of smoking-related disease	No	5604	14.4 (13.2, 15.5)	11.0 (10.0, 12.1)
	Yes	409	14.1 (10.1, 18.1)	10.5 (6.8, 14.1)

W6: Wave 6

[a]Weighted to US population using wave 6 cohort 4 all-wave longitudinal weights

[b]For race/ethnicity, groups categorized as "Others" were Hispanic, Black race alone, Asian race alone, and multi-racial

© 2025 Quach NE et al. *JAMA Network Open*.
