# Supplementary material for: Daily or Nondaily Vaping and Smoking Cessation Among Smokers
Source: JAMA Netw Open. 2025 Mar 5;8(3):e250089. doi: 10.1001/jamanetworkopen.2025.0089 (PMC11883493; doi:10.1001/jamanetworkopen.2025.0089)
Supplement: Supplement 2. — Data Sharing Statement [file jamanetwopen-e250089-s002.pdf]

Data Sharing Statement

Quach. Daily or Nondaily Vaping and Smoking Cessation Among Smokers. *JAMA Netw Open*. Published March 05, 2025. doi:10.1001/jamanetworkopen.2025.0089

Data
Data available: Yes
Data types: Deidentified participant data, Data dictionary
How to access data: The restricted Use file data are available at https://www.icpsr.umich.edu/web/NAHDAP/studies/36231
When available: The data can be accessed on the URL above after getting approved access (as written in the Additional Information section of this page below)

Supporting Documents
Document types: None

Additional Information
Who can access the data: Researchers can apply to access restricted-use files from the Population Assessment of Tobacco and Health (PATH) Study through the ICPSR Virtual Data Enclave (VDE) portal. To access the data, researchers must complete a Restricted Data Use Agreement
Types of analyses: Applications need to specify the purpose for the types of analyses that they plan to undertake
Mechanisms of data availability: With approval of a project and completion of a Restricted Data Use Agreement
